# Impact of postoperative isolated patellar chondromalacia on return-to-sport readiness, knee function, and strength following primary anterior cruciate ligament reconstruction: a retrospective comparative study

**DOI:** 10.3389/fphys.2026.1931981

**Published:** 2026-08-25

**Authors:** Alikemal Yazıcı, Ahmet Serhat Genç

**Affiliations:** 1 Department of Orthopedics and Traumatology, Faculty of Medicine, Samsun University, Samsun, Türkiye; 2 Department of Orthopedics and Traumatology, Samsun City Hospital, Samsun, Türkiye

**Keywords:** anterior cruciate ligament reconstruction, chondromalacia patella, muscle strength, return-to-sport readiness, single-leg hop test

## Abstract

**Background:**

The aim of this study was to investigate the association of isolated chondromalacia patella that developed during short-term follow-up after isolated primary anterior cruciate ligament reconstruction (ACLR) on pain, knee function, quality of life, muscle strength, and objective functional performance measures used in return to sport (RTS) assessment.

**Methods:**

In the study, 80 male patients who underwent primary ACLR using hamstring autograft were divided into two groups: those who developed chondromalacia in the first year postoperatively (n=40) and those who did not (n=40).Clinical assessment included the Visual Analog Scale (VAS), the International Knee Documentation Committee (IKDC) score, the Lysholm score, and the Tegner Activity Scale; physical function and quality of life were assessed using the Knee Injury and Osteoarthritis Outcome Score – the Knee Outcome Score-Physical Function (KOOS-PS), and the Short Form-12 Health Survey (SF-12) were used Functional performance was assessed using five different Single Leg Hop Tests (SLHT) and isometric dynamometer measurements of knee extensor and flexor muscle strength. Intergroup, inter-side, and group × side interactions were analyzed.

**Results:**

Pain levels were significantly higher chondromalacia group, whereas IKDC, Lysholm, KOOS-PS, and SF-12 physical component scores were significantly lower (p < 0.05). No significant differences were found between the groups in terms of Tegner activity level and SF-12 mental health component scores (p > 0.05). In objective assessments related to RTS, medial rotation hop test performance and knee extensor strength were significantly lower in the chondromalacia group (p < 0.05). In both groups, the operated limb demonstrated a significant performance deficit compared with the contralateral limb in all hop tests as well as in knee extensor and flexor strength (p < 0.001). In addition, group × side interaction analyses revealed significant differences associated with the presence of chondromalacia in SLHTs and knee extensor strength (p < 0.001).

**Conclusions:**

The development of isolated chondromalacia patella following ACLR is associated with increased pain, impaired knee function, reduced quality of life, weakened quadriceps performance, and inadequate objective RTS criteria. Because this degeneration is a significant prognostic factor that limits functional recovery, patellofemoral health and RTS criteria should be evaluated together during rehabilitation.

## 1 Introduction

The anterior cruciate ligament (ACL) is a key functional component of a healthy knee joint, and due to high functional demands, acute ACL tears are particularly common in young and athletic individuals (Jevremovic et al., 2024). ACL tear is characterized in the short term by pain, instability, and reduced range of motion due to joint effusion (Cimino et al., 2010; Hewett et al., 2013), and in the long term by the early onset and accelerated progression of osteoarthritic changes (Beynnon et al., 2005; Lohmander et al., 2007; Friel and Chu, 2013). Although meniscal and femorotibial lesions accompanying ACL tears are important determinants of functional outcomes after ACL reconstruction (ACLR) (Brophy et al., 2021; Randsborg et al., 2022) patellar osteochondral lesions are generally considered less significant in the literature. However, cartilage lesions have been reported to be associated with the long-term development of osteoarthritis (Li et al., 2011; Widuchowski et al., 2011). In particular, patellofemoral osteoarthritis has been shown to be common following ACLR performed with a hamstring tendon autograft, and this condition has been associated with more severe knee symptoms, including anterior knee pain, as well as reduced functional performance (Culvenor et al., 2016). Therefore, chondromalacia patella, which is frequently detected on magnetic resonance imaging (MRI) during short-term follow-up after ACLR, is of major clinical importance (Bahadır and Özkan, 2024).

Chondromalacia patella is a condition in which the cartilage on the surface of the patella softens and deteriorates; its most common and accurate diagnosis is based on arthroscopic findings (Levy et al., 2023). Patellar chondromalacia presents clinically as vague, diffuse pain in the front of the knee that is felt behind or around the patella (Jevremovic et al., 2024). Symptoms typically worsen with activities that increase reaction forces on the patellofemoral joint, such as climbing or descending stairs, squatting, or kneeling (Habusta et al., 2026). During a physical examination, a sensation, or sound of crepitus, is typically detected during knee flexion and extension; this is associated with fibrillation and roughness on the articular cartilage surface (Habusta et al., 2026). However, cartilage lesions and pain in the patellofemoral joint can trigger a neurophysiological protective reflex, leading to arthrogenic muscle inhibition (Rice and McNair, 2010; Eseoğlu et al., 2024). By inhibiting maximal voluntary contraction of the quadriceps muscle, this condition may lead to asymmetry in knee extensor strength and has the potential to directly affect patients’ shock absorption mechanisms as well as performance on return-to-sport (RTS) tests (Schmitt et al., 2012; Güzel et al., 2023; Genç et al., 2023). In the literature, it is well established that a limb symmetry index (LSI) of ≥90% should be achieved in tests assessing lower extremity function, such as single-leg hop tests (SLHTs), to ensure a safe RTS (Thomeé et al., 2011; Grindem et al., 2016; Yılmaz et al., 2025). In addition to all of this, the presence of chondromalacia patella following ACLR may affect clinical and functional outcomes. These undesirable clinical findings, which arise due to the presence of patellar chondromalacia, can affect LSI and may be a critical factor in the decision to RTS.

The primary aim of this study was to evaluate lower extremity functional strength as well as knee extensor and flexor strength in patients who developed chondromalacia patella during short-term follow-up after ACLR and to determine whether RTS criteria are met in patients with post-ACLR chondromalacia patella. It was hypothesized that the development of postoperative chondromalacia patella would not only be associated with worse pain, knee function, and quality of life, but also lead to marked impairment in muscle strength and functional performance, thereby making it more difficult to meet RTS criteria.

## 2 Materials and methods

### 2.1 Participants

A total of 80 male recreational-level athletes who underwent isolated primary ACL reconstruction using a single-bundle hamstring tendon autograft performed by the same orthopedic surgeon between January 2022 and April 2025 were included in the study. Preoperatively, the patellofemoral articular cartilage of all patients was evaluated using MRI, and only those with completely normal patellofemoral cartilage (Modified Outerbridge Grade 0) were included in the study. Based on routine MRI at the first postoperative year, patients were divided into two groups: those who developed isolated chondromalacia patella (Chondromalacia Group, n = 40) and those without evidence of chondromalacia (Control Group, n = 40). To maintain equal group sizes for a balanced mixed-design ANOVA which enhances robustness against potential violations of homogeneity of variance—40 patients were randomly selected from the eligible pool of 82 controls. Chondromalacia patella was graded using a modified MRI grading system based on the Outerbridge arthroscopic classification (Outerbridge, 1961; Shahriaree, 1985). An *a priori* power analysis was conducted using G·Power (version 3.1). Based on previous literature indicating a significant association between quadriceps strength deficits following ACLR and patellofemoral chondral lesions (Wang et al., 2015) knee extensor strength was defined as the primary outcome variable. Assuming a large effect size (Cohen’s d = 0.80), an alpha level of 0.05 and 80 per cent statistical power, at least 26 patients were required in each group. Consequently, it was deemed sufficient to include 40 patients in each group. The inclusion criteria were: Participation in recreational-level sports, undergoing unilateral primary ACL reconstruction, use of a hamstring tendon autograft, surgery performed by the same surgeon, and completion of a standardized postoperative rehabilitation program under the supervision of the same physiotherapist. Exclusion criteria were: A history of ACL revision surgery, prior knee arthroplasty, meniscal injuries requiring surgical intervention, multiligamentous knee injuries, focal osteochondral lesions, advanced tibiofemoral or patellofemoral osteoarthritis, or incomplete clinical or imaging data.

### 2.2 Study design

**Figure 1:**
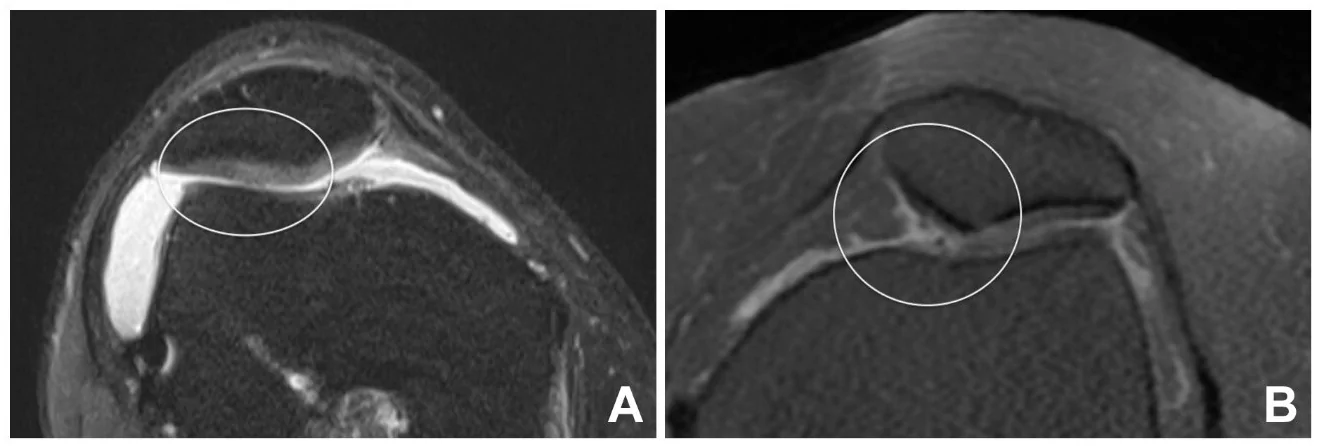
**(A)** Grade 1, **(B)** Grade 2 chondromalacia on axial MRI.

**Figure 2:**
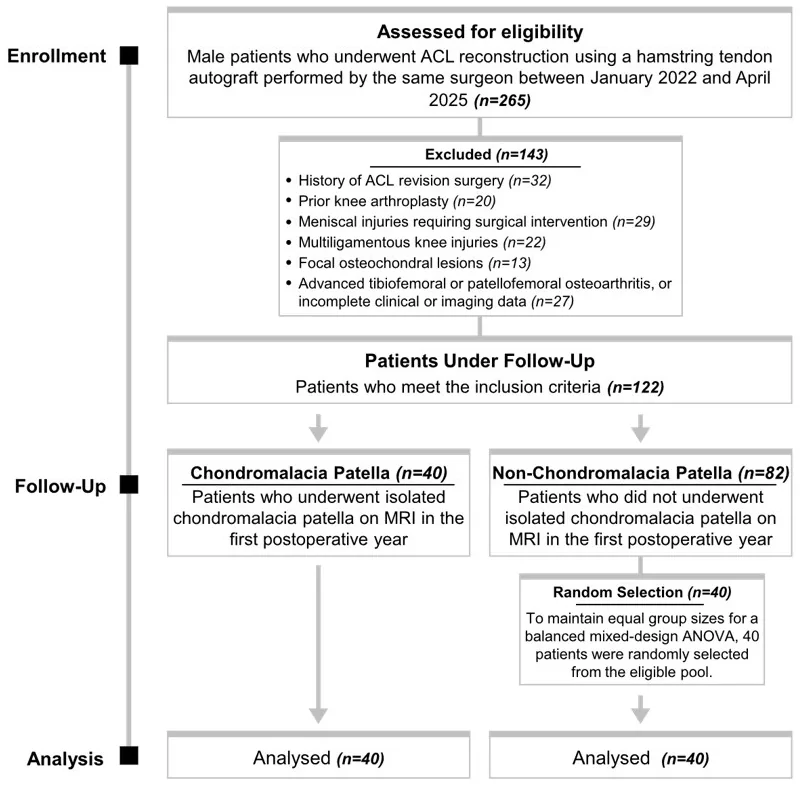
Flowchart.

This retrospective comparative study was conducted to evaluate the effects of isolated chondromalacia patella developing during short-term follow-up after isolated primary ACLR on pain, knee function, quality of life, muscle strength, and physical performance (**Figure 2**). Electronic medical records of the patients, including clinical evaluation results obtained during routine follow-up, physical performance test outcomes, and MRI findings, were retrospectively reviewed. The severity of patellar chondromalacia was universally graded using the Outerbridge Classification, as determined by a senior musculoskeletal radiologist via high-resolution MRI (**Figure 1**). All MRI examinations were performed using a standard 1.5-Tesla scanner utilizing axial, coronal, and sagittal T1-weighted, T2-weighted, and proton density fat-saturated sequences. Grade I was defined as softening and swelling of the articular cartilage without surface disruption (Habusta et al., 2023), whilst Grade II was defined as cartilage fragmentation and fissure formation affecting an area smaller than 1.3 cm (0.5 inches) in diameter (Marx et al., 2005). The orthopaedic and trauma specialist who carried out all physical assessments, clinical tests and data collection procedures was kept completely unaware of the MRI results and group distributions throughout the assessment process. Prior to analysis, all data were anonymized, and patient confidentiality was protected in accordance with the principles of the Declaration of Helsinki. The study was approved by the Non-Interventional Clinical Research Ethics Committee of Samsun University (Decision No: GOKAEK 2026/9/10) and was conducted in accordance with the principles of the Declaration of Helsinki. All clinical assessments, patient-reported outcome measures, functional performance tests, isometric strength measurements, and follow-up MRIs were conducted concurrently at the 12th postoperative month. In the short-term postoperative follow-up after ACLR, the Lysholm score, Tegner Activity Scale, International Knee Documentation Committee (IKDC) score, Visual Analog Scale (VAS), Knee Injury and Osteoarthritis Outcome Score–Physical Function Short Form (KOOS-PS), and SF-12 Health Survey were assessed. Additionally, functional performance was assessed using SLHTs, including the single-leg hop for distance (SH), single hop (SH), triple hop (TH), crossover triple hop (CH), medial side triple hop (MSTH), and medial rotation (90°) hop (MRH), while isometric knee extensor and flexor muscle strength was also measured.

### 2.3 Procedures

#### 2.3.1 Surgical technique and rehabilitation

All surgical procedures were performed by the same orthopedic surgeon using the arthroscopic anteromedial portal technique. The femoral and tibial tunnels were created based on the anatomical footprint. The femoral tunnel was created while preserving a 1–2 mm posterior cortical wall between the lateral intercondylar ridge and the posterior cortex, whereas the tibial tunnel was positioned at the native ACL footprint center. A quadrupled semitendinosus tendon autograft was prepared, and an adjustable-loop cortical suspension system was secured to both ends using No. 2 FiberWire^®^ sutures. A 12-mm cortical fixation system was used on the femoral side and a 21-mm cortical fixation system on the tibial side. While the graft diameter ranges from 8 to 10 mm, the graft length has been standardized to 6.5–7.0 cm. The tunnel diameters were determined based on the graft thickness, and final fixation was performed with the knee flexed at 30°. The same surgical technique and the same fixation method were used in all patients.

All patients underwent a standardized rehabilitation program administered by the same physical therapist following surgery. For the first 4 weeks, partial weight-bearing and the use of a cane were recommended. Knee range of motion was progressively increased in a controlled manner, with the aim of achieving full joint range of motion by the sixth postoperative week. During the rehabilitation process, muscle strengthening and proprioceptive exercises were gradually increased. The initiation of linear running and basic physical training, defined as Return to Physical Activity (RTPA), occurred at a mean of 6.6 to 6.8 months postoperatively. The decision to RTS was made between 9 and 12 months post-surgery, using a rigorous, multidisciplinary approach. Final RTS approval depended on patients meeting specific objective functional criteria; these included achieving a LSI of 90% or higher in all jump tests, achieving pain-free full range of motion, and complete elimination of fluid accumulation in the knee. Importantly, this decision-making process involved a multidisciplinary assessment where physical performance metrics were interpreted in conjunction with the orthopedic surgeon’s clinical evaluations and radiological assessments of the internal joint structures, not just functional and performance tests administered by physiotherapists and sports scientists. This ensured that RTS approval was only granted when both functional recovery and joint homeostasis were comprehensively achieved.

#### 2.3.2 Clinical evaluations

Lysholm, Tegner, and International Knee Documentation Committee (IKDC): The Lysholm score assesses symptoms of instability in the knee and other regions, the Tegner Activity Scale evaluates activity levels to determine readiness for return to regular activity or sport based on health status, and the IKDC score evaluates domains such as functional recovery, symptoms, and RTS (Tegner and Lysholm, 1985; Irrgang et al., 2001; Genç et al., 2023).

Visual Analog Scale (VAS): This scale is used to assess knee pain and is self-administered by patients. Patients indicate their pain level by marking a point on a 10-cm line ranging from “no pain” to “the worst pain imaginable.” Higher scores indicate more severe pain.

Knee Injury and Osteoarthritis Outcome Score—Physical Function Short Form (KOOS-PS): The KOOS-PS is a seven-item scale used to assess the difficulties people experience in daily activities related to knee health. All items are rated on a five-point Likert scale with scores ranging from 0 to 4 (none, mild, moderate, severe, extreme). The scale is scored on a range from 0 (no problem) to 100 (severe problem) and assesses how smoothly individuals are able to perform these activities.

SF-12 Quality of Life Survey (SF-12): The SF-12 is a shortened version of the SF-36 and is used to assess health-related quality of life. The scale consists of 12 items; the summary scores for the physical and mental components (PCS and MCS) are calculated and evaluated using a specific scoring algorithm. Scores are expressed on a 0 to 100 scale, with higher scores indicating better quality of life.

#### 2.3.3 Functional performance evaluation

**Figure 3:**
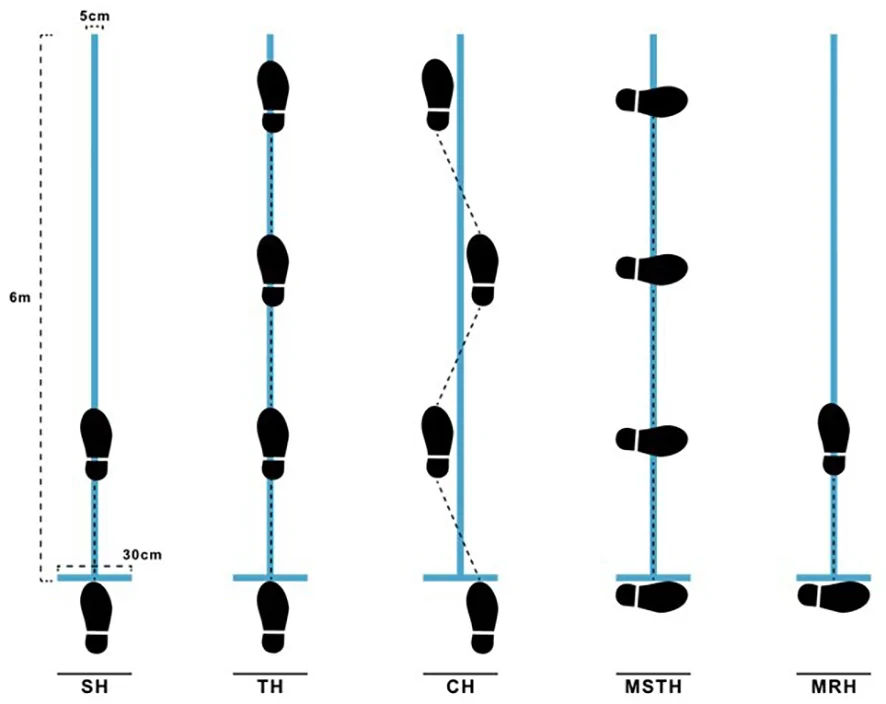
Single-leg hop tests.

Single-Leg Hop Tests (SLHTs): The SLHT battery, which is reported in the literature to have high test–retest reliability and is used to compare limb function and strength between the operated and non-operated sides in patients after ACLR, consisted of five different components: SH, TH, CH, MSTH, and MRH. The SLHT battery was administered to all participants (SH, TH, CH, MSTH, and MRH) in a fixed order. Testing always began with the uninjured contralateral limb. Participants were allowed to perform two practice trials for each test prior to the three recorded trials.These tests were performed along a straight 6-meter line with a width of 5 cm, starting from the designated point, and required each jump to begin on the tested limb and conclude with a controlled, balanced landing on the same limb (**Figure 3**). In SH test, the patient was instructed to stand on the test limb and perform a maximal forward jump in the sagittal plane to achieve the greatest possible distance in a single effort. In TH test, three consecutive maximal forward hops were performed on the same limb without pausing between jumps. In CH test, the patient started from the side opposite the tested limb relative to the reference line and performed three consecutive hops in a zigzag pattern, crossing the line with each jump, with each landing required to remain close to the line. During MSTH, participants stood in a lateral position with the medial aspect of the foot facing the line and performed three consecutive hops in the medial direction. In MRH test, they started from a similar lateral stance and were instructed to rotate 90° toward the medial direction in the air and land accordingly, without changing foot orientation prior to take-off. There was a 30-second break between the trials. The best recorded score was measured as the distance from the starting line to the participant’s heel at landing. Arm swing was not restricted during testing. If the patient lost balance, landed on a limb other than the starting limb, or performed an additional hop after landing, the trial was repeated (Kehribar et al., 2022; Güzel et al., 2023; Eseoğlu et al., 2024; Yılmaz et al., 2025). The LSI value, which gives the ratio between the opposite limbs, is calculated using SLHT distances via the formula ‘(operative/contralateral) × 100’.

#### 2.3.4 Isometric knee strength assessment

Knee extensor and flexor muscle strength were assessed using the ForceFrame strength testing system (Vald Performance Pty Ltd, Brisbane, Australia). Sensors with a sampling rate of 50 Hz were used in the data acquisition process. For knee extensor strength assessment, participants stood on the non-tested limb while the tested limb was positioned with the knee at 30° of flexion, with the distal tibia placed against the center of the dynamometer. Participants were instructed to maintain an upright posture and to avoid excessive forward or backward trunk lean. For knee flexor strength assessment, the setup was similar; with the knee positioned at 30° of flexion, the dynamometer was adjusted so that its center was in contact with the posterior aspect of the heel. During all tests, participants were strictly instructed to cross their arms over their chest to ensure trunk stabilization and prevent compensatory upper body movements. Participants were then instructed to extend the knee and press the posterior aspect of the lower leg against the pad for the required duration. The protocol consisted of three maximal voluntary contractions of five seconds each, with a 30-second rest period between repetitions. The mean of the three recorded trials was used as the final outcome measure, and all data were processed using the VALD ForceFrame digital platform (Wrona et al., 2023). Strength was analyzed as absolute peak force (N). The LSI value was calculated using the formula ‘(operative/contralateral) × 100’.

### 2.4 Statistical analysis

The data were analyzed using IBM SPSS Statistics Version 22.0 (IBM Corp., Armonk, NY, USA). Continuous variables were tested for normality using the Shapiro–Wilk test, and homogeneity of variances was assessed using the Levene test. Descriptive data were presented as mean ± standard deviation or as a percentage (%). An independent samples t-test was used to compare clinical outcomes between groups. The main effects and interactions between the lower extremity (operated/contralateral) and group (chondromalacia/control) were analyzed using a two-way mixed-design analysis of variance (mixed ANOVA) with respect to hop tests and isometric muscle strength. For the mixed ANOVA, the sphericity assumption was met automatically as the within-subject factor (limb) had only two levels. To control for the family-wise error rate across multiple functional hop tests, a Bonferroni correction was applied. For t-test analyses, Cohen’s d effect sizes and 95% confidence intervals were calculated. In the mixed-effects analysis of variance, effect size was reported as partial eta-squared (η²p). In all analyses, a p-value of <0.05 was considered statistically significant.

## 3 Results

The demographic and clinical characteristics of the study participants are presented in **Table 1**. No significant differences were observed between the chondromalacia and non-chondromalacia groups regarding age, height, body weight, BMI, time to RTPA, or follow-up duration (p>0.05). The mean time to RTPA was comparable between the non-chondromalacia and chondromalacia groups (6.8 ± 1.2 vs. 6.6 ± 1.4 months, p=0.656). Likewise, the mean follow-up duration did not differ significantly between the groups (12.52 ± 3.5 vs. 12.65 ± 3.2 months, p = 0.869). The mean time from injury to surgery for chondromalacia was 3.7 ± 1.6 and non-chondromalacia 3.9 ± 1.5 months, with no significant difference between the groups.Regarding the side of ACL reconstruction, right knee involvement was observed in 62.5% of patients in the non-chondromalacia group and 55.0% in the chondromalacia group, whereas left knee reconstruction was performed in 37.5% and 45.0% of patients, respectively. Among patients with postoperative patellar chondromalacia, MRI evaluation demonstrated Grade I chondromalacia in 9 patients (27.5%) and Grade II chondromalacia in 31 patients (72.5%).

**Table 1:** Demographics and clinical characteristics.

Variables	Non-chondromalacia Mean ± SD	Chondromalacia Mean ± SD	p
Age in years	26,95 ± 6.04	27.77 ± 4.73	0.499
Height in cm	181.97 ± 6.25	179.22 ± 4.85	0.153
Weight in kg	85.17 ± 10.73	83.37 ± 11.21	0.464
BMI in kg/m^2^	25.73 ± 3.18	26.28 ± 3.54	0.474
RTPA time in months	6.8 ± 1.2	6.6 ± 1.4	0.656
Follow-up time in months	12.52 ± 3.5	12.65 ± 3.2	0.869
Time from injury to surgery in months	3.7 ± 1.6	3.9 ± 1.5	0.848
ACL reconstruction side, n(%)			
Right	25 (62.5)	22 (55)	
Left	15 (37.5)	18 (45)	
MRI chondromalacia grade, n(%)			
Grade I	—	9 (22.5)	
Grade II	—	31 (77.5)	

SD standard deviation; BMI body mass index; RTPA return to physical activity; ACL anterior cruciate ligament; MRI magnetic resonance imaging..

**Table 2** presents a comparison of postoperative pain, knee scores, and quality of life across the groups. Statistically significant differences were observed in VAS (p=0.023, ES = 0.51), IKDC (p=0.009, ES = 0.60), Lysholm (p=0.003, ES = 0.69), KOOS-PS (p=0.036, ES = 0.48), and SF-12 Physical component scores (p<0.001, ES = 1.20). These results indicate that patients who developed chondromalacia exhibited poorer outcomes in terms of pain, knee function, and physical quality of life. In contrast, no statistically significant differences were found between the groups in terms of Tegner or SF-12 MCS (p>0.05). Notably, an effect size of 1.20 for SF-12 PCS indicates a large magnitude of difference between the groups regarding physical quality of life.

**Table 2:** The comparison of postoperative pain, knee scores, and quality of life.

Variables	Non-chondromalacia	Chondromalacia	t	p	ES	95% CI	
	Mean ± SD	Mean ± SD				LB	UB
VAS	3.33 ± 2.09	4.5 ± 2.39	-2.32	0.023*	0.51	-2.17	-0.17
IKDC	75.97 ± 13.80	67.97 ± 12.86	2.68	0.009**	0.60	2.06	13.94
TEGNER	6.42 ± 1.78	5.80 ± 1.52	1.679	0.097	0.38	-0.12	1.36
LYSHOLM	89.80 ± 8.97	82.75 ± 11.39	3.074	0.003**	0.69	2.49	11.61
KOOS-PS	5.77 ± 4.34	11.00 ± 14.90	-2.128	0.036*	0.48	-10.11	-0.35
SF-12 PCS	70.90 ± 12.14	57.38 ± 10.35	5.360	<0.001***	1.20	8.50	18.54
SF-12 MCS	67.54 ± 14.68	63.23 ± 10.57	1.506	0.136	0.34	-1.38	10.00

^*^ p<0.05, ^**^p<0.01, ^***^p<0.001. SD, standard deviation; ES, effect size; 95%, CI 95% confidence interval of the mean difference; LB, lower bound; UB, upper bound; VAS, visual analog scale; IKDC, international knee documentation committee; KOOS-PS, knee injury and osteoarthritis outcome score—physical function short form; SF-12 PCS, short form 12 health survey; SF-12 MCS, short form 12 health survey mental component summary.

**Figure 4:**
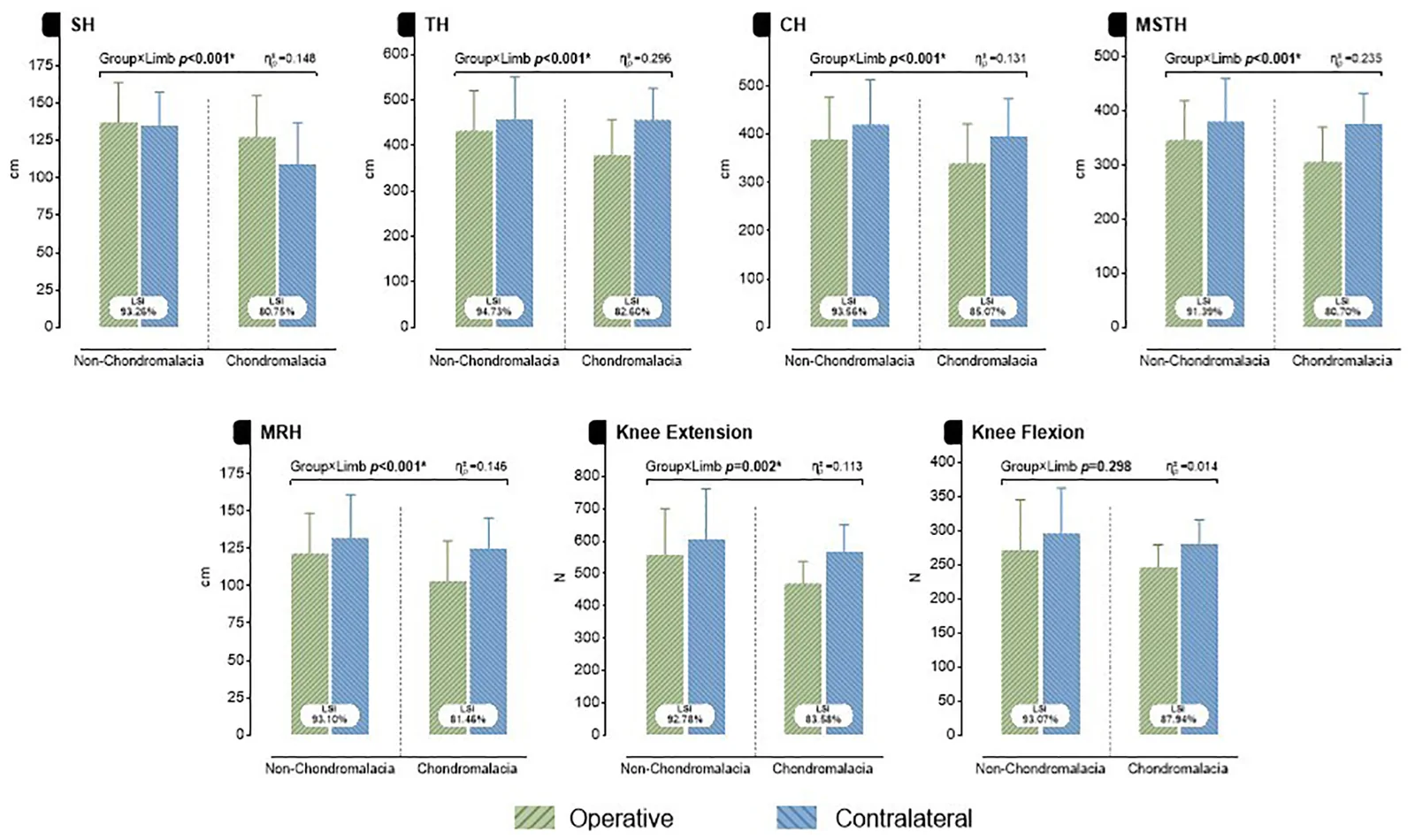
Comparison of single-leg hop tests and knee extensor and flexor strength between the chondromalacia and non-chondromalacia groups. *p<0.05, η²p partial eta square; SD standard deviation; LSI limb symmetry index; SH single hop for distance; TH triple hop for distance; CH crossover triple hop for distance; MSTH medial side triple hop for distance; MRH medial rotation (90°) hop for distance.

The comparisons of hop performance and knee muscle strength between groups are presented in **Figure 4**. Mixed-model ANOVA demonstrated significant main effects of limb for all hop performance tests and knee muscle strength measurements (all p < 0.001), indicating lower performance of the operated limb compared with the contralateral limb regardless of group. Regarding the main effect of group, significant differences were observed for MRH (p = 0.028, η²p = 0.060) and knee extension strength (p=0.013, η²p=0.076), with patients in the chondromalacia group demonstrating lower performance than those without chondromalacia. No significant group effects were identified for SH, TH, CH, MSTH, or knee flexion strength (all p>0.05). Significant group × limb interactions were identified for SH (p<0.001, η²p=0.148), TH (p<0.001, η²p=0.296), CH (p<0.001, η²p=0.131), MSTH (p< 0.001, η²p=0.235), MRH (p<0.001, η²p=0.146), and knee extension strength (p=0.002, η²p=0.113). These interactions indicate that the magnitude of side-to-side asymmetry was significantly greater in the chondromalacia group, rather than implying an overall between-group difference. In contrast, no significant interaction was observed for knee flexion strength (p=0.298). Based solely on descriptive data, without conducting statistical inference tests, LSI values for SLHTs and knee extension strength in patients with postoperative patellar chondromalacia tended to be lower than those in patients without chondromalacia. Descriptively, the most pronounced differences in LSI were observed in the TH (82.60% vs. 94.73%), MSTH (80.70% vs. 91.39%), MRH (81.46% vs. 93.10%) and the knee extension strength test (83.58% vs. 92.78%).

## 4 Discussion

The main finding of this study was that patients who underwent isolated ACLR using a hamstring tendon autograft and developed isolated chondromalacia patella during short-term postoperative follow-up demonstrated lower scores compared with those without chondromalacia in pain and quality of life assessments (VAS, IKDC, Lysholm, KOOS-PS, and SF-12 PCS), as well as reduced lower extremity strength, specifically in knee extensor strength and MRH. In addition, LSIs were also affected and were found to be lower in the chondromalacia group. The study findings demonstrated that, in the early postoperative period following ACLR, patients who developed chondromalacia patella experienced significantly higher pain levels, lower clinical knee scores, and adversely affected physical performance and quality of life compared with those without chondromalacia.

Despite successful ACLR, alterations in knee kinematics during the postoperative period and changes in patellofemoral joint loading may predispose to patellofemoral cartilage degeneration (Tashman et al., 2004; Culvenor et al., 2014). It has been reported that degenerative changes may occur early in the patellofemoral articular cartilage following ACL injury, and that these changes may contribute to the development of post-traumatic osteoarthritis in the long term (Li et al., 2011; Widuchowski et al., 2011; Culvenor et al., 2016; Culvenor et al., 2019). In particular, it has been reported that degenerative changes in the patellofemoral joint may occur following ACLR performed with hamstring tendon autografts, and that these changes may be associated with more severe knee symptoms, including anterior knee pain, as well as reduced functional performance (Culvenor et al., 2014). Chondromalacia patella, which is frequently detected following ACLR, exacerbates symptoms during activities that increase patellofemoral joint reaction forces, such as stair ascent/descent and squatting (Habusta et al., 2026). In our study, the significantly higher VAS pain scores observed in the chondromalacia group are fully consistent with the characteristic diffuse anterior knee pain associated with chondromalacia beneath or around the patella (Levy et al., 2023; Jevremovic et al., 2024). Moreover, the significantly worse KOOS-PS scores, which reflect difficulties in activities of daily living, along with lower IKDC and Lysholm scores, which indicate overall functional recovery, observed in the chondromalacia group may be interpreted as a direct clinical manifestation of increased biomechanical stress and pain (Tegner and Lysholm, 1985; Irrgang et al., 2001). The SF-12 PCS, which assesses health-related quality of life, was lower in the chondromalacia group compared with the non-chondromalacia group, whereas the SF-12 MCS and the Tegner activity score, which reflects return to regular activity and sport participation, were comparable between the groups. In our study, the largest between-group difference in patient-reported outcome measures was observed in the SF-12 PCS, suggesting that postoperative chondromalacia patella negatively affects not only knee-specific symptoms but also overall physical functioning in daily life activities. This suggests that patients maintain expectations of RTS, yet their physical performance, reflected by reduced LSI and knee extensor strength, may still be insufficient to meet these expectations.

Although the effects of meniscal, patellofemoral, and tibiofemoral cartilage lesions on pain, quality of life, and functional outcomes after ACLR have been investigated in the literature (Culvenor et al., 2016; Culvenor et al., 2019; Brophy et al., 2021), to our knowledge, there are limited studies that simultaneously evaluate the impact of isolated chondromalacia patella developing after isolated ACLR on pain, patient-reported outcomes, muscle strength, and functional performance. Therefore, this study contributes to the existing literature by jointly evaluating both subjective and objective functional outcomes in this specific patient population (Wang et al., 2015). In their assessment following ACLR, it was reported that greater than 80% recovery in quadriceps strength was associated with less severe patellar cartilage damage in short-term follow-up (Wang et al., 2015). In our study, significant differences were observed between the operated and contralateral limbs in SLHT performance as well as in knee flexion and extension strength. In the Group × Limb interaction analyses, the chondromalacia group demonstrated substantially lower SLHT performance compared with the non-chondromalacia group. Although we did not perform correlation or regression analyses to confirm direct causality, based on existing literature, we hypothesize that this difference in hop performance may be closely related to the significant deficit in knee extensor strength observed in the chondromalacia group. Furthermore, this finding suggests that chondromalacia patella developing after ACLR may increase functional limb asymmetry and negatively affect functional recovery of the operated limb. Pain and cartilage lesions in the patellofemoral joint can induce arthrogenic muscle inhibition, a neurophysiological protective mechanism that impairs maximal voluntary activation of the quadriceps muscle (Rice and McNair, 2010). Reduced quadriceps strength impairs shock absorption during the landing phase of hop tests, which may increase kinesiophobia and result in shorter jump distances (Schmitt et al., 2012; Trigsted et al., 2018; Culvenor et al., 2019; Güzel et al., 2023; Genç et al., 2023). In our study, knee extensor strength was considerably lower in the chondromalacia group compared with the non-chondromalacia group, and LSIs were also adversely affected. In the chondromalacia group, LSI values for hop tests remained in the 80–85% range, falling below the commonly accepted ≥90% threshold for safe RTS (Thomeé et al., 2011; Grindem et al., 2016). This may indicate the need for specific quadriceps strengthening modifications targeting the patellofemoral joint during ACL rehabilitation in these patients. Furthermore, caution is required when interpreting LSI values, as post-injury deconditioning of the contralateral uninjured limb may artificially inflate symmetry estimates, highlighting that LSI should not be utilized as a standalone criterion for RTS. In contrast, the similarity in Tegner activity levels between the groups suggests that self-reported activity status may not fully reflect objective functional recovery. In other words, although patients who developed chondromalacia patella reported similar activity levels, the reduced LSI values observed in objective functional assessments indicate that functional recovery in these patients did not reach the expected level. No significant difference was observed between the groups in knee flexion strength; this was an expected finding given that patellofemoral pathology primarily affects the extensor mechanism and that the same graft type was used in both groups, resulting in comparable hamstring strength outcomes. The inclusion of only patients who underwent ACLR using a hamstring tendon autograft represents a methodological strength of the study, supporting the interpretation that deficits in the extensor mechanism are strongly associated with patellofemoral pathology, namely chondromalacia.

From a clinical perspective, these findings suggest that postoperative chondromalacia patella should be considered in rehabilitation progression and RTS decision-making after ACLR. Although current guidelines recommend criterion-based and multidimensional RTS assessment, our results indicate that similar self-reported activity levels may coexist with persistent pain, knee extensor weakness, and suboptimal hop-test LSIs in patients with chondromalacia (Meredith et al., 2020; Kotsifaki et al., 2023). Therefore, RTS decisions should not rely solely on postoperative time or perceived activity level but should also include patellofemoral symptoms, quadriceps strength, and objective functional performance. Rehabilitation may require individualized load management and progressive knee- and hip-focused strengthening before progression to high-impact and sport-specific activities (Collins et al., 2018; Willy et al., 2019).

Our study has several limitations. First, the retrospective, archive-based observational design limits the ability to establish definitive causal relationships, and the exact temporal relationship between the onset of chondromalacia and the functional assessment cannot be definitively confirmed; therefore, our findings represent cross-sectional associations rather than temporal causal pathways. Second, the sampling method used for the control group may introduce potential selection bias, and there remains a possibility of residual confounding from unmeasured variables. Third, while preoperative and postoperative cartilage status was evaluated using MRI, MRI-based classification has inherent limitations regarding reliability and sensitivity for detecting early cartilage softening compared to a gold-standard assessment such as second-look arthroscopy. Furthermore, the inclusion of only young adult male participants limits the generalizability of the findings to female patients and other age groups. In addition, future prospective studies with longer postoperative follow-up periods should investigate the correlation between MRI-based grades of chondromalacia and objective performance deficits, which may provide further valuable contributions to the literature. Additionally, while our cohort comprised recreational athletes without work-related injuries, the lack of detailed records on specific sport types remains a limitation. Finally, future studies with larger and more balanced subgroups are needed to investigate the effect of lesion severity on functional recovery.

## 5 Conclusions

The presence of chondromalacia patella in the short-term follow-up after ACLR is associated with increased pain, reduced subjective quality of life, lower knee extensor strength, and suboptimal objective hop test performance. It is therefore important for clinicians to closely monitor signs of patellofemoral pain syndrome in the postoperative period and consider adapting rehabilitation protocols in the presence of chondromalacia. Finally, whether routine combined monitoring of patellofemoral cartilage status via MRI and quadriceps function can facilitate the development of individualized rehabilitation strategies and support more objective RTS decision-making remains a promising area for future prospective investigations.

## Data Availability

The data analyzed in this study is subject to the following licenses/restrictions: Data supporting the findings of this study are available through the corresponding author, but restrictions apply to the availability of these data used for the current study and are therefore not publicly available. However, data are available from the corresponding author upon reasonable request. Requests to access these datasets should be directed to Ahmet Serhat Genç, ahmetserhatgenc@hotmail.com.
